# A Five-Year Prospective Study of Diabetic Retinopathy Progression in Chinese Type 2 Diabetes Patients with “Well-Controlled” Blood Glucose

**DOI:** 10.1371/journal.pone.0123449

**Published:** 2015-04-07

**Authors:** Peiyao Jin, Jinjuan Peng, Haidong Zou, Weiwei Wang, Jiong Fu, Binjie Shen, Xuelin Bai, Xun Xu, Xi Zhang

**Affiliations:** 1 Department of Ophthalmology, Shanghai First People’s Hospital, Shanghai Jiao Tong University, Shanghai, 20080, China; 2 Department of Preventative Ophthalmology, Shanghai Eye Disease Prevention and Treatment Center, Shanghai, 200040, China; 3 Beixinjing Community Health Service Center, Shanghai, China; Zhongshan Ophthalmic Center, CHINA

## Abstract

**Purpose:**

To determine the progression rate and risk factors for diabetic retinopathy (DR) in Chinese type 2 diabetic patients who have reached the target hemoglobin A1c (HbA1c) level recommended by the American Diabetes Association.

**Methods:**

This was a 5-year community-based prospective study. The study population consisted of patients with type 2 diabetes with HbA1c less than 7.0%. Demographic information, systemic examination results and ophthalmological test results for each participant were collected. The outcome of this study was the progression of DR, which was defined as an increase in DR grade in one or both eyes at the final visit in comparison to the baseline status. The association between each potential risk factor and DR progression was studied.

**Results:**

A total of 453 patients with HbA1c less than 7.0% were included in the study group. In 146 patients (32.22%), DR developed or progressed during the five-year follow-up. Baseline HbA1c level was the only independent risk factor for DR progression (*p*<0.01, OR = 2.84, 95%CI: 2.11~3.82). The logistic regression function suggested that the possibility of DR progression increased fastest when baseline HbA1c increased from 5.2% to 6.4%. The 5-year DR progression rate in patients with baseline HbA1c less than 5.2%, between 5.2% and 6.4%, and over 6.4% were 19.62%, 24.41%, and 76.83%, respectively.

**Conclusions:**

To slow the progression of DR in Chinese patients with type 2 diabetes, more intensive glucose control is recommended.

## Introduction

In 2010, the age-standardized prevalence of diabetes in Chinese adults was 9.7%, representing at least 92 million Chinese diabetes patients[1]. A significant proportion of these patients were suffering from diabetic retinopathy (DR). Two studies from China found that the prevalence of DR was 37.1% in Beijing[2] and 43.1% in Handan[3]. During the last decade, our study group performed several epidemiological surveys in the Beixinjing community of Shanghai, China, and found a 25% prevalence of DR among diabetic residents[4,5]. Although many treatments are available, DR remains the most common cause of blindness among people 30 to 69 years old in several Western countries[6,7]. It is commonly believed that diabetic complications are exacerbated by hyperglycemia. Therefore, the American Diabetes Association (ADA) recommended in 2008 a glycosylated hemoglobin (HbA1c) goal for non-pregnant adults in general of less than 7.0% (53 mmol/mol) to reduce the microvascular and neuropathic complications of diabetes[8].

Since 2003, our study group has been helping diabetic residents in the Beixinjing community, Shanghai, China, to control hyperglycemia in order to prevent DR onset and progression[5]. However, after almost 10 years of observation, we found that DR still progressed in a number of patients with “well-controlled” hyperglycemia (HbA1c less than 7.0%, as recommended by the ADA). The “Action in Diabetes and Vascular Disease: Preterax and Diamicron ME Controlled Evaluation Study” (the ADVANCE study) also has suggested that intensive glucose control targeting HbA1c less than 6.5% did not significantly reduce the incidence and progression of retinopathy[9]. The key to DR control is to prevent it from happening or to treat it in an early stage. To achieve this target, it is necessary to identify the factors associated with DR progression in diabetes patients with “well-controlled” glucose. It is also essential to explore whether the ADA-recommended target HbA1c level is effective in reducing DR progression in a Chinese population.

The results reported here were based on a 5-year prospective study. The purpose of this study was to determine the progression rate of DR and the factors associated with it in a Chinese type 2 diabetic population whose hyperglycemia is “well-controlled” (HbA1c less than 7.0%, as recommended by the ADA) and to further explore whether the ADA-recommended target HbA1c level is effective in reducing DR progression in a Chinese population. Because the clinical features of diabetes differ among different races[6,10] and because most previous studies were conducted in Western countries[9,11], the results of this study revealed a number of previously unreported facts regarding DR progression in an Asian population.

## Methods

### Setting and participants

The Beixinjing community is located in the southwestern corner of the Shanghai urban district. The population of this community has remained generally stable over the past twenty years. In 1995, a residents’ health information database covering almost all residents was begun. This database has been updated annually by the Beixinjing Community Health Service Center [4].

The inclusion criteria for the present study were: diagnosed type 2 diabetes patients (according to the WHO definition[12]); more than 18 years old; willingness to accept a general physical examination once a year; willingness to control blood glucose with drugs or behavioral therapy under the guidance of doctors; and willingness to sign the informed consent. The exclusion criteria were suffering from severe systemic disease other than diabetes (such as severe heart/brain vessel disease or cancer), final stage (grade 4) DR in both eyes, or suffering from severe eye diseases other than DR (such as severe cornea opacity, severe cataracts, glaucoma, or retinal detachment).

Diabetes residents with “well-controlled” blood glucose (baseline HbA1c level less than 7.0%) and no invasive treatment history were included in the study group. The residents with higher glucose or previous invasive treatments were also observed at the start and the end of the study in order to obtain the 5-year DR progression rate in patients who did not reach the ADA recommended target glucose. The purpose of this study was to determine the progression rate and the associated factors for DR. As the clinical profile of DR suggests[13], most DR patients in this community with severe non-proliferative DR or proliferative retinopathy accepted photocoagulation, intraocular medicine injection or surgery to control the progression of DR. Because those treatments would influence the outcome of our study, only those patients with no obvious DR, mild non-proliferative DR or moderate non-proliferative DR and those with severe DR but who refused to undertake invasive treatments were included in the study group.

Apart from the baseline survey and the final survey, participants also received a general physical examination once a year. Fasting blood glucose was monitored by a capillary blood glucose determination twice a week during the follow-up period, and their HbA1c levels were measured once per year. If a participant’s blood glucose fluctuated largely, our research team recommended individualized anti-hyperglycemia drug or behavioral therapy to help control temporary hyperglycemia.

This study was conducted according to the tenets of the Declaration of Helsinki and was approved by the Institutional Review Board of Shanghai First People's Hospital, Shanghai Jiao Tong University. All of the participants were more than 18 years old. They understood the study protocol and gave written informed consent to this study.

### Anthropometric and Laboratory Measurements

The baseline survey was conducted in 2007. We acquired some of the participants’ information from the community health information database, including age, gender, duration of diabetes, age at diabetes diagnosis, occupation, education status, and general and ophthalmological medical history. The duration of diabetes was defined as the interval between the diagnosis of diabetes and the baseline examination. The height, weight, and systolic and diastolic blood pressure of the patients were measured by trained doctors. Blood pressure was measured in a supine position in the right arm, using a standard mercury sphygmomanometer. Hypertension was defined as systolic blood pressure ≥ 140 mmHg and/or diastolic blood pressure ≥ 90 mmHg. The body mass index (BMI) was calculated as weight (kg)/height^2^ (m^2^). According to the guidelines released by Chinese Ministry of Health, the definition of obesity was a BMI≥28[14]. Laboratory tests included serum triglycerides, total cholesterol, serum creatinine, urinary microalbumin and HbA1c. Serum creatinine, total cholesterol and triglycerides were measured using an enzymatic assay. Urinary microalbumin was analyzed by nephelometry. HbA1c was measured by ion exchange chromatography. A serum triglyceride≥1.7 mmol*L^-1^ was defined as hyperlipidemia and a serum total cholesterol≥5.2 mmol*L^-1^ was defined as hypercholesteremia. Microalbuminuria ≥ 30 mg/L and serum creatinine≥104 μmol*L^-1^ suggested renal dysfunction. All control values were consistent with the standards recommended by the Shanghai Clinical Testing Center.

### General Eye Examinations and Retinopathy Assessments

Eye examinations were conducted during the baseline survey (in 2007) and the final survey (in 2012). Visual acuity was tested by ETDRS tables with a 300 lux light. The eyelid, conjunctiva, cornea, iris, and lens were examined by a slit-lamp microscope (YZ-5, Liuliu Medical Instrument Company, Suzhou, China). The participants were screened for DR by a standardized protocol, which has been described in our previous paper[15]. The patients’ vitreous and fundus were examined by a direct ophthalmoscope, and the posterior pole was photographed with a non-mydriatic funduscopic camera (CR-DGI, Canon, Tokyo, Japan). Two 45-degree digital retinographs were obtained per eye. The retinographs were taken using the technique described in the EURODIAB study[16], with one centering on the macula and the other nasal to the optic disk. Two trained readers read the retinographs independently and classified the DR grade according to the well-accepted “International Clinical Diabetic Retinopathy Disease Severity Scale”[17]. The data are presented as 5 DR grades: no obvious DR (grade 0); mild non-proliferative DR (grade 1); moderate non-proliferative DR (grade 2); severe non-proliferative DR (grade 3); and proliferative retinopathy (grade 4). The two assessors were masked during the reading procedure. Any unresolved disagreements between the two assessors were referred to the group leader (H.Z.) for arbitration. Once DR was diagnosed by our screening examinations, the patient underwent another series of examinations in our hospital, including optical coherence tomography and fundus fluorescein angiography, to confirm the diagnosis and the severity scale. A sample of 80 retinal photographs (with and without retinopathy) was graded again to assess the validity. Overall, there was a high degree of agreement in the assessment of retinopathy with respect to internal and inter-observer reliability (k1 = 0.874 and k2 = 0.869).

The main outcome assessed was DR progression, which was defined as an increase in the DR grade in one or both eyes at the final visit compared with the baseline status. Participants with DR progression were classified into the progress group, and the others were categorized in the stable group. If a participant received surgery, intraocular medicine injections or photocoagulation treatment for DR during the 5-year follow-up period, he/she was classified into the progress group.

### Statistical Analysis

Two staff members independently input all of the data into an ACCESS database. SAS (version 9.3 SAS Institute, Cary, NC) was used for all statistical analyses. Descriptive information for each of the variables was derived and the distribution was assessed. Baseline characteristics were presented as the mean ± standard deviation for continuous variables and as rates (proportions) for the categorical data. For normally distributed continuous data, t-tests were used to compare means between groups, and for non-normally distributed continuous data, the Kruskal-Wallis H test was used. Categorical variables were compared with the Chi-square test. From the univariate logistic analyses, variables with p values under 0.5 were considered for entry into multiple logistic regression. Stepwise multiple logistic regression analysis was used to determine whether potential risk factors (including age, gender, diabetes duration, diabetes onset age, occupation, education level, BMI, baseline HbA1c, follow-up HbA1c, high blood pressure, serum triglycerides, total cholesterol, serum creatinine and urinary microalbumin) were associated with DR progression (inclusion value = 0.05 and exclusion value = 0.05). Matlab software was used to analyze the regression function. All reported *p* values are two-sided. Statistical significance was defined as *p*<0.05.

## Results

There were 811 residents in the Beixinjing community with type 2 diabetes in 2007, of which 3 had grade 4 DR in both eyes, and 13 had severe systemic or ocular disease other than DR. Of the remaining 795 diabetic patients, 453 patients with “well-controlled” glucose and a history of no invasive treatment were included in the study group. Although no missing cases occurred during the five years of follow-up study, one subject’s baseline blood pressure and serum creatinine data went missing. There was no evidence that these missing data might influence the accuracy of our analysis.

At the time of the baseline survey in 2007, the youngest participant was 20 years old, while the oldest was 87 years old. The years of education of the participants ranged from 0 to 20. The age at first diabetes diagnosis ranged from 9 to 83 years old. In 3 participants, diabetes was diagnosed within one year, while the longest diabetes duration was 33 years in a 74-year-old participant. The BMIs ranged from 16.7 to 35.6. All systolic blood pressures were lower than 140 mmHg, while the mean diastolic blood pressure was 80.5 mmHg. All participants’ microalbuminuria were in the normal range (under 30 mg/L). The serum creatinine levels ranged from 50.6 μmol*L^-1^ to 103 μmol*L^-1^, the blood triglycerides ranged from 0.5 mmol*L^-1^ to 2.9 mmol*L^-1^,^.^ and the total cholesterol ranged from 3.0 mmol*L^-1^ to 7.0 mmol*L^-1^. At the time of the baseline survey in 2007, the vision of the 453 participants ranged from hand movement to 20/20. Mild cataracts were found in 154 patients. Compared with the study group patients, those patients without glucose control were older, and had an earlier mean onset age, longer mean diabetes duration, and higher mean glucose (all p<0.05). The participants used anti-hyperglycemia drugs, including metformin, sulfonylureas, meglitinides, glitazones and insulin injections. Participants who also had high blood pressure continued their antihypertensive therapy during follow-up, using common drugs, such as diuretics, β-adrenoceptor blocking drugs, angiotensin-converting enzyme inhibitor (ACEI), angiotensin II receptor antagonist (ARB), and calcium channel blocking drugs. Participants with hyperlipemia or hypercholesterolemia took drugs such as statins to lower their lipid levels. Many patients also used calcium dobesilate drugs to improve microcirculation. However, most of them could not provide us with the exact dosage of their drugs. The general information for the participants is summarized in Table 1.

**Table 1:** Characteristics of the participants at the time of the baseline survey in 2007 and during the next 4-year follow-up period

	Total	Progress Group	Stable Group	P value
Number of participants	453	146 (32.23)	307 (67.77)	
Age (year)	66.66±15.21	68.32±14.86	65.87±15.33	0.02
Male Gender	166 (36.64)	56 (38.36)	110 (35.83)	0.60
Above high school	232 (51.21)	76(52.05)	156(50.81)	0.50
Diabetes onset age (year)	58.16±15.69	60.05±15.55	57.26±15.70	0.03
Diabetes duration (year)	8.49±6.08	8.27±6.29	8.60±5.98	0.36
Obesity	80 (17.66)	24 (16.44)	56 (18.24)	0.64
High blood pressure	197(43.49)	70(47.95)	127(41.37)	0.42
Hypercreatinemia	15 (3.31)	6 (4.11)	9 (2.93)	0.51
Hyperlipidemia	192 (42.38)	64 (43.84)	128 (41.69)	0.67
Hypercholesteremia	256 (56.51)	81 (55.48)	175 (57.00)	0.76
HbA1c at baseline (%)	5.61±0.78	5.99±0.92	5.43±0.62	<0.01
HbA1c<5.2%	158(34.88)	31 (21.23)	127 (41.37)	<0.01
5.2≤HbA1c<6.4	213(47.02)	52 (35.62)	161 (52.44)	
HbA1c≥6.4%	82(18.10)	63 (43.15)	19 (6.19)	
HbA1c in 2008(%)	5.74±0.44	5.71±0.43	5.75±0.45	0.36
HbA1c in 2009(%)	5.94±0.43	5.94±0.44	5.93±0.42	0.88
HbA1c in 2010(%)	5.95±0.42	5.96±0.41	5.95±0.42	0.79
HbA1c in 2011(%)	5.70±0.35	5.69±0.36	5.71±0.35	0.62

Among the 453 study group participants, 146 patients had DR progression during the 5-year follow-up (DR progression rate = 32.23%). In the 342 patients without glucose control, 173 had DR progression during the follow-up period (DR progression rate = 50.58%). The 5-year DR progression rate of the patients with well-controlled glucose was significantly lower than those without such control (*p*<0.01). Among the study group participants, the 146 patients with DR progression were classified into the progress group, and the remaining 307 participants were classified into the stable group. The baseline characteristics of the two groups are compared in Table 1, and the DR grades of all of the study group participants in the baseline survey and the final survey are reported in Table 2. Patients with different DR grades between their two eyes are classified by the eye with the higher grade. In the three grade 4 DR patients, grade 4 DR was only observed in one eye.

**Table 2:** Diabetic retinopathy grades of the worse eye of 453 participants in the baseline survey in 2007 and the final survey in 2012.

grade in 2007	grade in 2012					
	0	1	2	3	4	Total
0	89	46	20	11	6	172
1	73	98	33	9	6	219
2	16	20	9	2	0	47
3	5*	4	2	0	1	12
4	1†	1†	0	0	1	3
Total	184	169	64	22	14	453

*: subjects received photocoagulation during the follow-up

†: subject received surgery during the follow-up

Compared with subjects of the stable group, patients with DR progression had a higher mean baseline HbA1c level (*p*<0.01), but the mean HbA1c levels of these two groups in subsequent annual follow-up examinations were not significantly different (*p*>0.05), as shown in Table 1. Although statistically the progressed participants also had an older mean age (*p* = 0.02) and a younger mean onset age (*p* = 0.03), the marginal difference had little clinical meaning (Table 1). There was a highly significant positive association of DR progression with baseline HbA1c in the univariate analyses (*p*<0.01), while the other factors were not significantly related (*p*>0.05). Through univariate logistic regression, participants’ age, diabetes onset age, diabetes duration, blood pressure, baseline HbA1c and follow-up HbA1c levels entered multivariate logistic analysis (p<0.5). Multivariate logistic stepwise regression further suggested that only baseline HbA1c was independently related to DR progression(*p*<0.01, OR = 2.84, 95%CI: 2.11~3.82), as expressed by the following equation: ln [p ∕ (1-p)] = 1.08*HbA1c (%) – 6.93, where p is the probability of DR progression.

**Fig 1:**
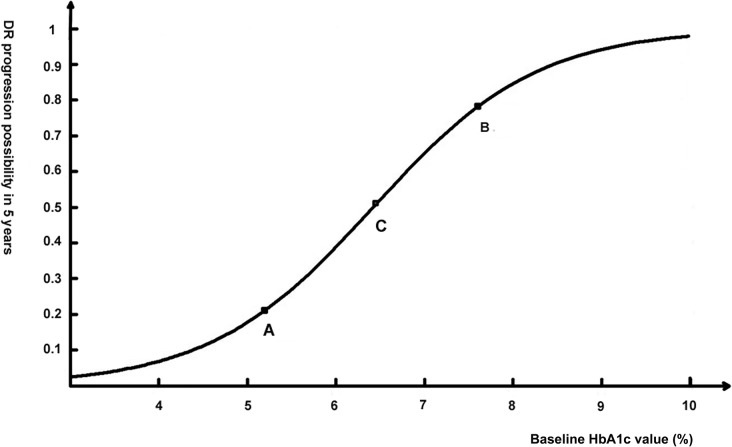
Logistic regression function curve: the association between initial HbA1c level and DR progression. Two maximum curvature points, A (5.2, 0.2) and B (7.6, 0.8), and one inflection point, C (6.4, 0.5), are labeled.

Matlab software was used to calculate the regression function. According to the function, when the baseline HbA1c is 7.0%, DR grade will progress in 65.25% patients in 5 years. There were two maximum curvature points, A (5.2, 0.2) and B (7.6, 0.8), and one inflection point (also referred to as the sym-center), C (6.4, 0.5), on the regression function curve (Fig 1)[18]. The maximum curvature point is the turning point between slow change and rapid change. Thus, point A indicated that once baseline HbA1c is above 5.2%, the increase in the probability of DR progression with increasing HbA1c speeds up, while point B indicated that the increase in the probability of DR progression slows down when baseline HbA1c is above 7.6%. The inflection point is the turning point between the concave and the convex regions. This also means that when a patient’s baseline HbA1c is 6.4%, there is a 50% possibility that his DR grade will progress in 5 years. The data showed that the 5-year DR progression rate of patients with baseline HbA1c less than 5.2%, between 5.2% and 6.4%, and greater than 6.4% were 19.62%, 24.41% and 76.83%, respectively (Table 1).

## Discussion

In this study, the 5-year progression rate of DR is not low in Chinese type 2 diabetic patients with HbA1c under control and below 7.0%. Thus, we believe that the achievement of the recently accepted hyperglycemia control target (HbA1c <7.0%) does not satisfactorily halt DR progression in Chinese type 2 diabetic patients. Our results suggested that the higher the initial HbA1c level, the higher the possibility of DR progression. To reduce DR progression, controlling glucose to a lower target level in the early period of diabetes is recommended.

The ADA recommends that the HbA1c level in diabetic patients should be controlled to less than 7.0%, and they also emphasize an incremental benefit to further lowering the HbA1c in selected individual patients to reach values as close to normal (<6.0%) as possible under the premise of no significant hypoglycemia[8]. Our research results provided substantial new evidence regarding the efficacy of these recommendations. Although significantly fewer patients with HbA1c less than 7.0% had DR that progressed than those patients who did not reach the target level, the progression rate was still not low. From the DR prevention perspective, we suggest that type 2 diabetic patients with no severe systemic complications such as severe heart / brain vessel diseases or cancer should control their blood glucose to a lower level under the premise of no severe side effects. For the patients in our study, an HbA1c target less than 5.2% might be ideal; however, it is premature to establish a target value. Additional prospective studies in different populations and of side effects should be conducted. Although it has been reported that intensive diabetes therapy has long-term beneficial effects on the risk of cardiovascular disease in type 1 diabetes patients[19], other research has indicated that strict glycemic control (HbA1c less than 6.0%) leads to large vessel damage and an increase in mobility[20]. Thus, for patients already at a high risk of circulation problems, a surrogate target of HbA1c less than 6.4% was suggested. Reviewing former research, we found that our result is consistent with some other large clinical trials. Recently, “The Action to Control Cardiovascular Risk in Eye Study” (the ACCORD study) reported that intensive glycemic control (target HbA1c <6.0%) reduced the rate of DR progression in type 2 diabetic patients[11]. However, the ADVANCE study found that intensive glucose control (target HbA1c<6.5%) did not reduce DR incidence and progression in type 2 diabetic patients[9]. Because those studies are randomized controlled trial (RCT) studies observing specific diabetic patients with strict controls on the study population, our population-based study is closer to the real life situation and provides stronger evidence.

Our results showed a strong association between baseline HbA1c level and DR progression. However, there was no significant difference between the progress group and the stable group in HbA1c levels during the follow-up period. We speculated that this might result from the “metabolic memory” phenomenon. In 1990, Roy and his colleagues coined the term “metabolic memory” to describe the hypothesis that systemic metabolic imbalance may continue to develop in patients who no longer have hyperglycemia[21]. Two well-known studies have supported this theory in Western populations, “The Diabetes Control and Complications Trial Study” (the DCCT study) in type 1 diabetes[22] and the “United Kingdom Prospective Diabetes Study” (UKPDS) in type 2 diabetes[23]. They found that among patients with similar glucose levels, DR often occurred in those with higher initial HbA1c. Significant evidence has indicated that hyperglycemia leads to oxidative injury, microthrombi formation, cell adhesion molecule activation, leukostasis and cytokine activation. The combination of cytokines, including vascular endothelial growth factor, insulin-like growth factor-1, angiopoetin-1 and -2, stromal-derived factor-1, fibroblast growth factor-2 and tumor necrosis factor, leads to further long-term retinal damage. This may explain the prolonged damage caused by high glucose and the prolonged benefit of strict glycemic control[24]. To date, no conclusive theory has been presented to describe the mechanism of metabolic memory. However, it is clear that in addition to a lower target glucose level, type 2 diabetic patients should reduce glucose to this target level as soon as possible to avoid a negative metabolic memory.

Some studies indicated that the advantage of lower initial blood glucose disappears in teenage type 1 diabetes patients[25,26] however, we found that “metabolic memory” is not affected by age. In this study, DR progression was not related to blood pressure or BMI, which is consistent with the results of many previous reports[9,11,25], but in disagreement with a study conducted in an Alberta First Nations population[27]. We also found that DR progression is not related to gender, disease duration, or age at diabetes onset. Furthermore, DR progression was found not to be related to initial blood lipids, which is inconsistent with the results of the ACCORD study[11]. We speculated that all of these differences resulted from differences in study populations, as most previous studies on intensive glycemic treatment are RCT studies in non-Asian populations, while the participants in this study were enrolled from the general Chinese population.

The 5-year DR progression rate of patients with "well-controlled" blood glucose in our study was 32.23%, while the 4-year DR progression rate reported in the ACCORD study[11] and the ADVANCE study[9] was 7.3% and 26.80%, respectively, and the 6-year DR progression rate reported in the UKPDS study[28] was 24.60%. Compared with Western RCT studies, the DR progression rate in our study is much higher, which might result from population differences. Other studies have suggested that the Asian population is more sensitive to high glucose than Caucasian patients and is thus more likely to develop DR[6,29]. In addition, the participants in the UKPDS study were newly diagnosed diabetic patients, and the median of diabetes duration of the participants in the ADVANCE study was 6 years, which are both shorter than our study population. Moreover, the target HbA1c level for intensive glucose control in the ACCORD study and the ADVANCE study were 6.0% and 6.5%, respectively, which is lower than our inclusion criteria, HbA1c less than 7.0%. Another possible reason is that the outcome measurement used in our study was the "International Clinical Diabetic Retinopathy Disease Severity Scale", while the UKPDS study, the ADVANCE study and the ACCORD study all used the "Early Treatment of Diabetic Retinopathy Study" (ETDRS) scale change as the outcome. Different outcome measurements could lead to difference in progression rates.

Despite the high incidence rate, cases of DR regression were quite common in our study, especially in patients with “well-controlled” glucose and mild baseline DR. Spontaneous diabetic retinopathy reversal has been noted by many ophthalmologists and reported by some researchers[30–33]. It has been reported that background retinopathy is not merely a process of cumulative increase in the number of microaneurysms but also reflects a dynamic balance between microaneurysm formation and disappearance. In patients with good glucose control, microaneurysm formation showed a decreasing trend [34,35]. We speculated that this phenomenon could explain the reversal in our patients. Long-term maintenance of controlled glucose leads to a lower rate of new microaneurysm formation, and the existing background microaneurysms eventually disappeared. Another possible explanation for the high regression rate found in the present study is that the drugs our patients used to control hypertension and to improve microcirculation may also promote DR regression. Previous studies have suggested that ACEI therapy has an additional protective effect in promoting the regression of DR[36–38], and it has been reported that pycnoyenol prevents the progression of retinopathy and partially recovers visual acuity[39,40].

This study suffers from several specific limitations in addition to the weakness inherent in all population-based investigations. First, the conclusions of this study may only apply to Chinese type 2 diabetes patients. Second, the retinographs were taken centering on only the macula and the optic disk; thus, retinopathy outside of these areas might have been missed. Third, this research was only a prospective case-control study. The results of the study need to be verified by longer term, multi-race, and multicenter cohort or RCT studies.
